# Evaluation of Clinical Decision Rules for Bone Mineral Density Testing among White Women

**DOI:** 10.1155/2013/792831

**Published:** 2013-01-10

**Authors:** Michael E. Anders, Lori Turner, Jeanne Freeman

**Affiliations:** ^1^Department of Respiratory and Surgical Technologies, College of Health Related Professions, University of Arkansas Medical Sciences, 4301 W. Markham, Little Rock, AR 72205, USA; ^2^Department of Health Science, College of Human Environmental Sciences, University of Alabama, P.O. Box 870311, Tuscaloosa, AL 35487, USA; ^3^Department of Physical Education, Health, and Recreation, College of Humanities and Social Sciences, Western Washington University, 516 High Street, Carver Gym 102, Bellingham, WA 98225-9067, USA

## Abstract

*Background*. Osteoporosis is a devastating, insidious disease that causes skeletal fragility. Half of women will suffer osteoporotic fractures during their lifetimes. Many fractures occur needlessly, because of inattentiveness to assessment, diagnosis, prevention, and treatment of osteoporosis. *Study Purpose*. Study Purpose. To evaluate the discriminatory performance of clinical decision rules to determine the need to undergo bone mineral density testing. *Methods*. A nationally representative sample from the Third National Health and Nutrition Examination Survey consisted of 14,060 subjects who completed surveys, physical examinations, laboratory tests, and bone mineral density exams. Multivariable linear regression tested the correlation of covariates that composed the clinical decision rules with bone mineral density. *Results*. Increased age and decreased weight were variables in the final regression models for each gender and race/ethnicity. Among the indices, the Osteoporosis Self-Assessment Tool, which is composed of age and weight, performed best for White women. *Study Implications*. These results have implications for the prevention, assessment, diagnosis, and treatment of osteoporosis. The Osteoporosis Self-Assessment Tool performed best and is inexpensive and the least time consuming to implement.

## 1. Introduction

 Osteoporosis is a devastating, insidious chronic disease that causes skeletal fragility. Half of women and one-eighth of men will suffer osteoporotic fractures during their lifetimes [1–3]. The occurrence of disability following osteoporotic hip fracture exceeds that of stroke, heart disease, or cancer and often leads to a profound forfeiture of independence [4–8]. Among people one year after the occurrence of hip fracture, seven in 10 are unable to walk independently, eight in 10 are unable to perform instrumental functions such as driving or shopping, and one-third of those residing in a nursing home had been living in a residence other than a nursing home prior to hip fracture [9–11]. Sequelae of hip fractures, such as pneumonia and pulmonary embolism, are frequently lethal [1]. One in four people die during the year subsequent to hip fracture, and one-third of these deaths are attributable to hip fracture [9, 12, 13]. Indeed, falls are the leading cause of injury-related death among people aged 65 years and older in the US [14]. Though men have a lower incidence of hip fractures than women, they are twice as likely to die in the year afterward [15].

 People are also living longer. The World Health Organization's (WHO) health profile of the United States reports the life expectancy for women to be 81 years based on 2010 data [16]. Consequently, with the projected growth in the 65 and older population, the number of hip fractures will increase. Osteoporosis plays a role in 90 percent of all hip fractures and 45% of all adults who present with hip fracture have had a prior fracture [17]. Many of these fractures occur in women who have been undiagnosed and untreated for osteoporosis and many of these fractures can be prevented [18].

 The dismal rates of morbidity and mortality associated with osteoporosis extract an enormous toll on our economy. Annual direct costs of osteoporotic fractures in the United States were estimated at $17 billion dollars in 2005 with cumulative costs over the next two decades to exceed $474 billion [18, 19]. As “baby boomers” age, the osteoporosis “time bomb” may explode. In the Framingham Study, the risk of hip fractures proliferated with each successive generation [20]. Modeling of future incidence and prevalence rates of osteoporotic fractures indicates that by 2025, fractures are projected to grow by more than 48% to greater than 3 million [21]. 

 Osteoporosis screening with bone mineral density (BMD) testing can lead to timely diagnosis, effective medical management, and prevention of fractures [22–25]. The National Osteoporosis Foundation established guidelines for patient selection for BMD testing based on identification of major risk factors [26] and refined them in 2008 [27]. Some family physicians were aware of but did not use clinical practice guidelines for osteoporosis [28]. These clinicians criticized existing guidelines as too complex. Hence, the transformation of these guidelines from theory into practice was lacking. Primary care providers often fail to recognize patients at risk for this insidious disease and thereby fail to prescribe treatment [29, 30]. Alternately, family physicians appealed for more succinct, practical guidelines and expressed enthusiasm for a clinical decision rule for BMD testing [28]. 

 Several clinical decision rules for referral for BMD testing have been proposed [31–36]. Among these, the Osteoporosis Self-Assessment Tool (OST), comprised of age and weight as the only variables, is the most succinct [33]. Studies have demonstrated the selectivity of the OST as a clinical decision rule for referral for BMD measurement, and the Surgeon General's report on bone health and osteoporosis recommended that clinicians consider using a chart version of the OST, or “Chart OST” [33, 37–42]. Two other relatively simple clinical decision rules for referral for BMD testing are the Osteoporosis Risk Assessment Instrument (ORAI), which is comprised of age, weight, and estrogen use, and the Age, Body Size, No Estrogen (ABONE), which is comprised of age, weight, and estrogen or oral contraception use [31, 36]. Cadarette, et al. [43] reported that the ORAI outperformed the National Osteoporosis Foundation guidelines, but the ABONE was less sensitive. 

 The purpose of this study was to evaluate the discriminatory performance of the OST, ORAI, and ABONE as clinical decision rules for referral for BMD testing in a nationally representative sample.

*Theoretical Framework.* Clinical decision rules are evidence-based criteria to assist health care providers in making decisions about screening, diagnosis, treatment, or prognosis [44]. Such rules are based on patient history, physical examination, and diagnostic tests. Essential characteristics of valid clinical decision rules are generalizability and practicability [45]. Sensitivity, specificity, positive and negative predictive values, likelihood ratios, and the area under the receiver operator characteristic curve (ROC) are measures of predictive power. 

 Even a clinical decision rule with high predictive power will be ineffective if it lacks acceptance by health care providers. Simplicity and practicability are essential characteristics. The OST includes only two variables, age and weight; it is practical, because primary care providers routinely collect these data and a chart of the index is available for women. The ORAI and the ABONE include age, weight, and estrogen use. 

## 2. Methods

 The Department of Health and Human Services (DHHS), Centers for Disease Control and Prevention (CDC), designed and conducted the Third National Health and Nutrition Examination Survey (NHANES III). Data collection methods included structured surveys by trained interviewers, physical examinations, and diagnostic tests. Participants aged 2 through 17 years completed the Household Youth questionnaire; participants aged 17 or older completed the Household Adult questionnaire. Interviews were conducted in either English or Spanish. Survey questions elicited demographic, socioeconomic, dietary, and medical history data. Physical examination teams operated in mobile examination centers and consisted of physicians, dentists, dieticians, medical technologists, radiological technologists, sonographers, health interviewers, home examiners, and coordinators. The DHHS implemented exhaustive quality control standards that minimized threats to internal validity.

 The DHHS employed a stratified, multistage random sampling design. Primary sampling units consisted of 2,812 counties, parishes, and cities in the US [46]. After stratification, the sample included 81 primary sampling units at 89 survey locations widespread across the US In three stages, the survey questionnaire was completed by or for 13,944 individuals aged two months to 16 years and 20,050 individuals aged 17 years or greater [46]. The overall random sample was 33,994 from 39,696 eligible individuals for a response rate of 85.6 percent, which minimized nonresponse bias [46, 47]. Of these, 30,818, or 90.7 percent, completed a follow-up physical examination [46, 47]. The DHHS oversampled minorities, older people, and children, to increase the statistical power for analyzing these data, and provided sampling weights for each stratum. Exclusion criteria were (a) institutionalized civilians, (b) noncivilians, and (c) residency in the nonconterminous states, Alaska, and Hawaii.

 These data collection and sampling methods resulted in cross-sectional data that were valuable for both descriptive and analytical purposes.

### 2.1. Study Sample

 The sample consisted of 14,060 noninstitutionalized males and females, aged 20 through 90 years and greater, for whom BMD data were available from NHANES III. The sample included 1.719 non-Hispanic White females. 

### 2.2. Data Management

 The investigators used the Statistical Export and Tabulation System software (revision 805, July 2000) developed by the CDC, National Center of Health Statistics, to query and extract NHANES III data. The data files for the Household Adult questionnaire, the physical examination, and the laboratory from the CDC, National Center of Health Statistics CD-ROM, Series 11, No. 1, No. 1A, and No. 2A (revised October, 1997), were exported to respective Excel files. The data were then (1) cross-referenced, (2) matched by subjects using their unique identifying numbers, (3) merged into a single Excel file, and (4) imported into an SPSS file. The investigator stored the Excel and SPSS files on a drive that resided on a secure server. The Information Technology Department at the university archived this server on a daily basis. Only the primary investigator had access to the data files. The study employed SPSS, version 13.0 for Windows, statistical software to calculate and present statistics. 

## 3. Results

Demographic and anthropometric data were calculated and presented as means and standard deviations or proportions. The investigator tabulated the prevalence rates of the BMD *T*-scores ≤−2.5, ≤−2.0, and ≤−1.0 by OST risk categories, ethnicity, and gender.

 Using a liberal selection threshold of *P* < .25 in the univariable logistic regressions, the variables, “lack of physical activity” and “calcium intake,” were excluded from the full model multivariable logistic regression (see Tables 1 and 2). At an alpha =.01, the variables, “age,” “lack of estrogen therapy,” and “current smoker” increased the odds of a BMD < the NOF treatment threshold, and “weight” decreased the odds in the full and reduced models; alcohol was retained in the final model, because when it was subtracted the difference in the beta for lack of estrogen was greater than 20 percent. Variables excluded from the full model were retested in the reduced model and lacked significance. The Hosmer-Lemeshow goodness-of-fit test, *P* = .26, demonstrated adequate model fit. Interaction and collinearity were absent. 

### 3.1. Clinical Decision Rules in Older White Women

**Figure 1:**
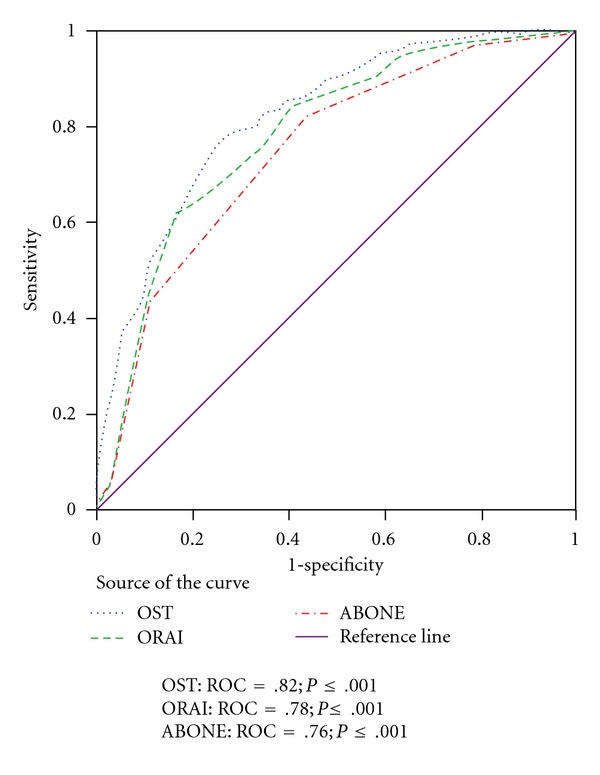
ROC: *T*-score ≤ −2.0, non-Hispanic White women, age ≥ 50 years.

 In older White women the OST and ORAI were strong predictors of osteoporosis; the ABONE was less effective (see Figure 1). The clinical decision rules demonstrated a similar pattern of performance in the prediction of the NOF treatment threshold. The performance of the three clinical decision rules declined and demonstrated less variance in the prediction of osteopenia. 

## 4. Discussion

 In a publication in the JAMA series, *User's Guide to the Medical Literature*, McGinn et al. [45] outlined three essential components of valid clinical decision rules: (a) appropriate method of development, (b) validation among a wide range of samples, and (c) ample predictive power. 

The development of each of the OST, ORAI, and ABONE was the result of multivariable regression analyses. In the development of the ABONE clinical decision rule, Weinstein et al. [48] employed multivariable logistic regressions for osteoporosis of the total hip, femur neck, and spine. Their results indicated that increased age was a risk factor for osteoporosis; increased weight and a history of estrogen intake of least six months, in the form of either estrogen therapy or birth control pills, were protective factors. Age > 65, weight < 63.5 kg, and history of estrogen use each account for one point in the ABONE score; the cut score for referral for BMD testing is a score greater than or equal to two. Thus, estrogen therapy accounts for a one-third of the possible points in the ABONE indices. In comparison to the OST and the ORAI, the relatively lower contribution of age and weight in the scoring system likely accounts for the lower diagnostic accuracy of the ABONE demonstrated in these study results. 

 Cadarette et al. [31] developed the ORAI using multivariable logistic regression, with the NOF treatment threshold, −2.0 standard deviations below the mean for young adult women, at either the femur neck or the spine as the dependent variables. Their study results demonstrated that age, weight, and current estrogen therapy were associated with osteoporosis at both measurement sites. In the ORAI scoring system, age and weight account for 93 percent of the possible points, and either an age of 65 years or greater or weight less than 60 kg would result in a recommendation for referral for a BMD test, regardless of current estrogen therapy. This heavy weighting of age and weight helps to explain the relatively close approximation of the diagnostic accuracy of the ORAI to that of the OST demonstrated in the study results. 

 Koh et al. [33] developed the OST using linear regression, with femur neck BMD *T*-score as the dependent variable in Asian women. Age and weight were among several independent variables in the final regression model. However, the researchers dropped the other variables from the clinical decision rule due to their relatively minor contribution to the diagnostic accuracy of predicting osteoporosis. The minor contribution of hormone replacement therapy may be a result of a low rate of estrogen use among Asian women [49]. Results of the current study corroborated that age and weight were the predominant risk factors for a BMD at or below the NOF threshold for treatment and correlates of BMD. Hence, these study results supported the development method of the OST clinical decision rule as appropriate. 

 Among the logistic regression final models for the NOF treatment threshold among White women, only the final model included independent variables other than age and weight. In addition to age and weight the final model included lack of current estrogen therapy and current smoker as statistically significant independent variables. The inclusion of current estrogen corroborated the final model of the logistic regression for the development of the ORAI by Cadarette et al. [31], in which white women composed 95 percent of the sample. In a sample of Asian women, the study results of Koh et al. [33], too, included current estrogen in the final regression model. However, in their development of the OST, the researchers omitted current estrogen as a variable, because it made only a minor contribution to the diagnostic accuracy and applied only to a subset of women [33]. The inclusion of “current smoker” in the final model corroborated the determination that smoking is a major risk factor for osteoporosis in the evidence-based NOF clinical practice guidelines, which was based on studies with samples of predominantly Caucasian women [26]. 

 The present study provides further evidence of validation of the OST in various samples of Caucasian women. These studies of the OST included the following samples: (a) Geusens et al. [40] studied samples of predominantly white women in the US and the Netherlands; (b) Richy et al. [41] studied a sample of white Belgian women; (c) Cadarette et al. [37] studied a sample of Canadian women, which, presumably, predominantly consisted of Caucasian women; and (d) Rud et al. [50] studied a sample of predominantly Caucasian Danish women. 

 The current study corroborated the findings of previous studies of the discriminatory performance of the OST in Caucasian women [37, 40, 41, 50]. The OST demonstrated strong discriminatory performance in the prediction of osteoporosis: ROC = 83 percent, 95 percent CI: 81 percent, 85 percent; sensitivity = 95 percent, 95 percent CI: 93 percent, 97 percent; and specificity = 41 percent, 95 percent CI: 38 percent, 43 percent. In comparison, previous studies reported the following ROC values for osteoporosis: (a) Koh et al. [33]: 85 percent; (b) Geusens et al. [40]: 85 percent; (c) Richy et al. [41]: 81 percent; and (d) Cadarette et al. [37]: 82 percent.

### 4.1. Implications

 The study results had several implications. Among the decision rules, the OST demonstrated the best discriminatory performance, which was strong for white women. Hence, clinical application of the OST could help to minimize false negative findings, undiagnosed and untreated cases of osteoporosis, and devastating hip fractures. Furthermore, the OST was (a) the simplest indices, with only age and weight as variables, and (b) the most practical indices, because age and weight are commonly available data in clinical settings.

 The results helped to address each of the three essential components of a valid clinical decision rule, as outlined by McGinn et al. [45]: (a) the development method by regression analyses appeared to be appropriate; (b) it appeared to be applicable in a wide range of samples, perhaps even universally applicable to women 50 years of age and older, and; (c) it demonstrated strong and excellent predictive power. 

 Furthermore, the OST is relatively simple and practical, making it attractive for adoption in clinical practice. Only age and weight, routinely available data, are needed. Graphic representation of the OST calculation on a chart is therefore relatively easy. Such a chart can enable health care providers and patients alike to assess the risk of osteoporosis. 

 An evidence-based systematic review suggested that clinical decision rules improved physician performance [51]. Hence, with broad generalizability and robust predictive power in combination with simplicity, the OST can potentially improve physicians' assessment of patients' need to undergo BMD testing. The US Surgeon General punctuated the critical need for health care providers to adequately assess osteoporosis [38]. With adequate assessment, health providers can in turn initiate appropriate therapy and educate patients about preventive measures to avoid unnecessary devastating fractures and the associated degradation of health-related quality of life. 

### 4.2. Significance of the Study

 The study had implications for the prevention, assessment, diagnosis, and treatment of osteoporosis. The Surgeon General highlighted the development of strategies to identify the need to undergo BMD tests as a key research agenda for the future [38]. 

 Osteoporosis is an insidious, underdiagnosed, and undertreated disease. The National Osteoporosis Foundation guidelines for referral for BMD testing are complex and difficult to transform into clinical practice [26, 27]. Succinct clinical decision rules have practical applications. However, clinical decision rules require validation among a wide range of samples. To enhance generalizability of the OST, the study sought to validate these clinical decision rules in a representative national sample. 

## Figures and Tables

**Table 1:** Full model logistic regression: white females, age ≥ 50 years.

*n* = 1,537	*β*	S.E.	Wald	*P*	OR (95% CI)
Age (per 10 years)	.619	.069	81.06	<.001	1.86 (1.62, 2.12)
Weight (per 10 lbs.)	−.382	.031	150.40	<.001	.68 (.64, .73)
Weight loss	.085	.148	.327	.567	1.09 (.123, 2.59)
Lack of estrogen therapy	.579	.191	9.19	.002	1.78 (1.23, 2.59)
Chronic disease	.081	.134	.365	.545	1.09 (.83, 1.41)
Current smoker	.400	.142	7.95	.005	1.49 (1.13, 1.97)
Alcohol	−.327	.201	2.65	.103	.72 (.49, 1.07)
Serum Vitamin A	−.005	.004	1.46	.227	1.0 (.99, 1.0)
Serum Vitamin C	.099	.126	.62	.432	1.10 (.86, 1.41)
Serum Vitamin E	.000	.000	1.38	.241	1.0 (1.0, 1.0)

**Table 2:** Reduced model logistic regression: white females, age ≥ 50 years.

*n* = 1,719	*β*	S.E.	Wald	*P*	OR (95% CI)
Age (per 10 years)	.632	.064	97.41	<.001	1.88 (1.66, 2.13)
Weight (per 10 lbs.)	−.392	.027	214.03	<.001	.68 (.64, .71)
Lack of estrogen therapy	.671	.182	13.58	<.001	1.96 (1.37, 2.79)
Current smoker	.372	.137	7.38	.007	1.45 (1.11, 1.89)
Alcohol	−.379	.195	3.78	.052	.69 (.47, 1.0)
